# lncRNAs in development and disease: from functions to mechanisms

**DOI:** 10.1098/rsob.170121

**Published:** 2017-07-26

**Authors:** M. Joaquina Delás, Gregory J. Hannon

**Affiliations:** 1 Watson School of Biological Sciences, Cold Spring Harbor Laboratory, New York, NY 11724, USA; 2 Cancer Research UK Cambridge Institute, Li Ka Shing Centre, University of Cambridge, Cambridge CB2 0RE, UK; 3 New York Genome Center, 101 6th Ave, New York, NY 10013, USA

**Keywords:** long non-coding RNAs, development, cancer

## Abstract

Differential expression of long non-coding RNAs (lncRNAs) during differentiation and their misregulation in cancer highlight their potential as cell fate regulators. While some example lncRNAs have been characterized in great detail, the functional *in vivo* relevance of others has been called into question. Finding functional lncRNAs will most probably require a combination of complementary approaches that will greatly vary depending on their mode of action. In this review, we discuss the different tools available to dissect genetically lncRNA requirements and how each is best suited to studies in particular contexts. Moreover, we review different strategies used to select candidate lncRNAs and give an overview of lncRNAs described to regulate development and cancer through different mechanisms.

## 1. Introduction

Regulated gene expression is the basis for the extensive variety of cell types our bodies generate from the same set of DNA instructions. Specific gene programmes are transcribed in particular cells providing them with their molecular identity and the protein products that underlie their functions. Together with coding genes, thousands of long non-coding RNAs (lncRNAs) are also expressed in a cell-type-specific manner during differentiation and in certain cancers. This has been extensively reported in many organisms and cell types [1–4], yet demonstrating that these molecules play functional roles has not been easy.

The now ever-expanding catalogue of lncRNAs first became apparent from efforts to annotate the functional features of the human genome, which showed that the vast majority of the genome was transcribed [5]. Currently, lncRNAs are defined as capped transcripts longer than 200 nucleotides, which coincides with the cut-off for many RNA extraction protocols [6]. They can be spliced and, in most of the published studies, are also polyadenylated. LncRNAs have little or no coding potential, although some do bind to ribosomes [7–9]. They were originally described to have equivalent chromatin features to protein-coding genes [10]. However, more recent work has highlighted differences in the abundance of particular histone marks [11,12] and splicing efficiency [12,13] between lncRNAs and coding genes, as well as subsets of lncRNAs that differ in their chromatin signatures [4,14].

Hundreds of thousands of lncRNAs have been annotated in different species and tissues [15]. Of those, only a handful have been shown to be critical for organism development [16–18] or cancer progression [19], and the mechanisms by which they act have been established in just a few cases. For some, biochemical partners have been carefully identified, yet *in vivo* evidence for their function is missing or questions have been raised regarding the relevance of the previously reported mechanisms of action [20,21]. Bridging this gap is essential for building a solid body of knowledge of how lncRNAs function in cell fate choices and the mechanisms by which they act. In this review, we will focus on the different strategies to select and identify functional lncRNAs and some mechanistic examples of lncRNA acting in differentiation and cancer.

## 2. Cell-type-specific expression of long non-coding RNAs: cause or consequence?

The cell-type-specific expression observed for lncRNAs has provoked much excitement, as it implies that they might function during cell fate decisions. In accord with this notion, deregulation of lncRNAs has also been widely observed across human cancers [22–25]. Such disease-associated expression changes suggested a potential role for these lncRNAs in driving cancer or at least contributing to maintaining an aberrant transcriptional landscape.

### 2.1. Long non-coding RNA expression during development

Differential expression of lncRNAs has been reported between regions of the mammalian brain [26,27], and lncRNA dynamics have been analysed in more detail during corticogenesis [28]. Several studies have shown differential expression of lncRNAs in *in vitro* differentiation models of haematopoiesis [29,30] and in freshly isolated cell populations [31,32], as well as during mammalian adipogenesis [33,34]. This tissue and cell-type-specific regulation is observed across species, including during development of zebrafish [2], *Caenorhabditis elegans* [3], and even during the life cell cycle of our close unicellular relative, *Capsaspora owczarzaki* [4].

If lncRNAs are to regulate key developmental genes, a very appealing possibility is that they do so in *cis*. Correlated expression of lncRNAs and their neighbouring genes has been reported in embryonic stem (ES) cell differentiation to endoderm [35] and to embryoid bodies [36], as well as in human B and T cell lineages [37]. However, neighbour correlation is not a special property of lncRNA–gene pairs, as the expression levels of neighbouring genes are often correlated. This is thought to be due to shared regulatory elements affecting each neighbour [38] and to the general neighbourhood or chromosomal domain around them [39,40].

This leaves us with several possibilities to consider, and a myriad of experimental challenges to distinguish between them. The genomic location of lncRNAs and their neighbouring regulatory elements could determine their cell-type-specific expression, with the RNA being a mere by-product of the regulatory mechanisms already in place [41]. On the other hand, lncRNAs could be critical for expression of those developmental genes, orchestrating chromatin changes by specific RNA–protein interactions, or by increasing the local concentration of transcriptional machinery regardless of the actual RNA sequence transcribed. A general mechanism by which lncRNA transcription in specific cell types could reorganize nuclear architecture, and thus contribute to the new transcription landscape, has even been proposed [15].

### 2.2. Long non-coding RNA misregulation in cancer

Along the same lines, cancer-specific lncRNA expression could simply be a by-product of aberrant gene expression in cancer. However, genetic mutations can directly affect lncRNA expression, with the lncRNAs themselves playing a causal role in specific scenarios. LncRNA *CCAT2*, for example, encompasses a cancer-associated SNP. The risk allele correlates with a higher expression of the lncRNA, which in turn promotes proliferation in colorectal cancer [42]. This lncRNA is part of the 8q24.21 region, where many cancer-associated mutations and amplifications have been reported. Several disease-associated SNPs and translocations including the lncRNA *PVT1* have drawn researchers' attention to this very complex locus [43]. One of these amplifications is that of *PVT1* and its neighbouring gene *c-MYC*. In breast cancer models *PVT1* RNA levels correlate with MYC protein, yet *PVT1* and *c-MYC* are not always co-amplified. This suggests that amplification of this lncRNA alone (even without MYC) can promote tumorigenesis in breast cancer by increasing MYC expression [44], which has been proposed to occur by protein stabilization. However, it is unlikely that this is the only mechanism of action for this lncRNA as this multi-exonic transcript encoding over 20 different isoforms is itself under the control of c-MYC and harbours multiple microRNAs within its locus [45].

While these are a few well-characterized examples, a much greater number of functional studies will be required to tease apart collaterally expressed lncRNAs from those with important roles both in development and in cancer.

## 3. Finding functional long non-coding RNAs

Many experimental strategies have been used to dissect genetically lncRNA requirements in differentiation and cancer. Powerful in their own ways, each of these techniques has its own drawbacks. Therefore, a combination of complementary approaches will probably be required to reveal the biological impact of lncRNAs. The choice of approach also strongly depends on the biological question, whether it is the identification of lncRNAs important for a differentiation or disease process, the study of specific types of regulatory mechanisms—*cis* versus *trans*, or an in-depth analysis of a particular lncRNA.

### 3.1. Different tools for different questions

The main consideration is that, in a particular lncRNA locus, the act of transcription itself could be key to establishing or maintaining the chromatin state of the surrounding area, while, in this scenario, the actual sequence of the RNA would be irrelevant. Or the RNA itself could be the functional unit, having some sequence-dependent interactions with proteins, RNAs or DNA elements. It could even be that both these mechanisms apply for the same locus. Therefore, it is very important to understand each experimental set-up and what it tells us about each particular lncRNA.

**Figure 1.**
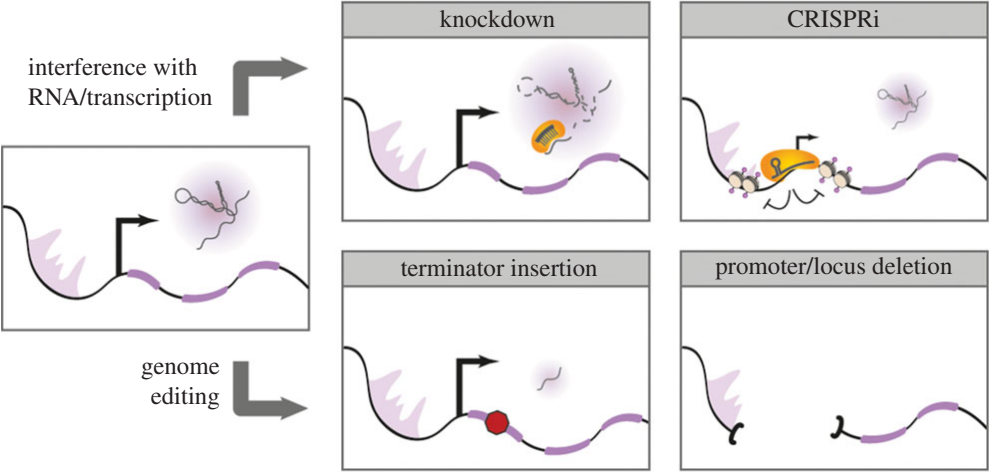
Different approaches for disrupting lncRNAs. Methods such as knockdown and CRISPRi affect the RNA itself or reduce the transcription of the lncRNA. Knockdown can be achieved in a variety of ways (siRNA, shRNA, LNA, ASO). CRISPRi is most efficient if Cas9 is fused to repressor domains (e.g. KRAB). These methods can also be transient. Insertion of an early terminator sequence or complete deletion of the locus or promoter are achieved via genome engineering and are non-reversible.

Several studies have taken advantage of RNA interference (RNAi) approaches, either transduced shRNAs or transfected siRNAs [46,47]. This strategy has been coupled with a phenotypic readout, such as viability or differentiation, to identify lncRNAs where the RNA molecule itself is important (figure 1). However, many worry about potential off-target effects (though this is no different from shRNA studies with protein-coding genes). There are additional concerns regarding the difficulty of knocking down lncRNAs that are chromatin-associated versus cytoplasmic, given that small RNA loading into the RISC complex takes places in the cytoplasm. While there is some evidence for differences in knockdown efficiency depending on subcellular location [48], this concern would apply only to lncRNAs that are never exported to the cytoplasm. LncRNAs that function in the nucleus but in *trans* could very well be exported just like other RNAs and then re-imported. Undoubtedly, the main advantage of knockdown is that it allows for high-throughput screens that could yield a list, though potentially incomplete, of lncRNAs with functions in the phenotypic assay of our choice.

To circumvent possible subcellular localization biases, other researchers have taken advantage of alternative knockdown techniques, independent of the RNAi machinery, that do not require processing in any specific cell compartment. Morpholinos targeting splice junctions or conserved regions have been used in zebrafish for identification of functional lncRNAs *in vivo* [16]. Locked nucleic acids (LNAs) have also been used in mammalian cells [49,50]. Both of these approaches rely on annealing a synthetic nucleic acid to the lncRNA and blocking its function or its splicing. Antisense oligos (ASOs) are another alternative that takes advantage of RNase-H activity. ASOs have been used in a variety of systems, both delivered to cultured cells and administered *in vivo* in mice [19,51]. Several ASOs have now been approved for clinical use and, although their targets so far have been coding genes, this opens up the path towards therapeutic targeting of lncRNAs. These approaches, although incredibly useful because they exclusively target the RNA, can only be deployed where these molecules can be injected or otherwise delivered. This would not allow for pooled high-throughput screening and misses out on the advantages of genetically encoding knockdown, which can be conditionally induced for *in vivo* studies.

The ultimate proof of functionality is a genetic knockout. They allow for the study of *in vivo* function and reduce the possibility of off-target effects. Discrepancies between the hypothesized mechanisms for some lncRNAs based on the *in vitro* data and the absence of or very mild phenotypes observed in knockout animals [20,52–54] have resulted in scepticism regarding broad regulatory roles for lncRNAs [55].

Two recent examples of lncRNA knockouts emphasize how, in some cases, phenotypes might be more context-specific than anticipated. *Malat1* is a very abundant lncRNA that localizes to nuclear speckles. Although it was hypothesized that this RNA was required for speckle or paraspeckle formation and maintenance and regulated alternative splicing through interaction with SR proteins [56], three independent mouse knockout models showed that *Malat1* was dispensable for viability [52–54]. Furthermore, *Malat1* was shown not to be required for nuclear speckle formation and its deletion did not affect SR protein phosphorylation [54]. However, when crossed with the MMTV-PyMT mouse model of human breast cancer, *Malat1* deletion impaired tumour progression as evidenced by a severe reduction in metastatic burden [19].

*LincRNA-EPS* was shown to have an anti-apoptotic role and be required for red blood cell development in tissue culture models of erythroid development [57]. The knockout mouse model for this lncRNA showed no defects in blood development. However, *LincRNA-EPS* controls expression of immune response genes in macrophages and proved essential for the animals to respond to endotoxin challenge [21].

Although their molecular mechanisms still remain to be elucidated, these RNAs are representative examples of how, just as for coding genes, some lncRNAs could play roles under particular stress or disease conditions. This could potentially be the case for other lncRNAs, whose proposed roles have met with controversy, such as *HOTAIR*, where knockout animals seem to be viable and healthy [20,58].

When removing a DNA locus to generate a knockout—especially when dealing with large deletion—any phenotype observed could be due either to loss of an encoded RNA or to deletion of DNA sequences that might include regulatory elements. For this reason, full transcript knockouts can be combined with complementary strategies to dissect roles of RNA from those of DNA elements. The possibilities of genome engineering are not limited to full locus knockout, but instead allow more subtle modifications, such as polyadenylation (poly-A) signal insertions for premature termination (figure 1). Taking advantage of CRISPR/Cas9, a recent study looked at whether a set of 12 lncRNAs regulated the expression of their neighbouring genes in *cis*. Although expression defects were observed upon promoter knockout for five lncRNAs, only one had the same effect when a poly-A signal was inserted, suggesting that only the regulatory elements in the DNA surrounding the promoter and not the RNAs were required for these *cis* effects [59]. An equivalent mechanism has been proposed to explain the differences between knockout and poly-A insertions for the lncRNA *Lockd* and its neighbour gene *Cdkn1b* [60].

Two independent models for *Fendrr* knockout showed that it was required for mouse development [17,18]. Interestingly, the phenotypes differed, one being embryonic lethal with a presumed requirement for lateral plate mesoderm [17], while the other was perinatal lethal [18]. These differences could be the consequence of the distinct genetic strategies, one being a triple polyadenylation insertion and the other one a whole gene replacement, emphasizing the need for complementary approaches that distinguish between DNA and RNA elements.

The scenario is substantially more complicated when the lncRNA and its target gene have overlapping transcripts. *Airn* overlaps in antisense with the imprinted gene *Igfr2*. Through a series of polyadenylation cassette insertions, it was shown that transcriptional termination of *Airn* only leads to *Igfr2* de-repression when the non-coding transcript no longer overlapped the *Igfr2* promoter. This work concluded that transcription of *Airn*, rather than the final transcript, is responsible for promoter silencing [61]. Combinations of promoter, exon knockouts and termination signals created using CRISPR/Cas9 have helped dissect the relationship between *Haunt* (also known as *linc1547* or *linc-Hoxa5*) and the *HoxA* locus. Knockdown, termination or deletions of the first exons lead to increased expression of *HoxA* genes during retinoid acid-induced differentiation of ES cells, supporting a repressive role for *Haunt* at the *HoxA* genes. However, deletion of the whole *Haunt* locus prevents expression of *HoxA*, presumably due to deletion of some regulatory DNA elements required for *HoxA* induction [62].

While more accessible owing to the advancements in genome engineering, mouse model generation is still not amenable to high-throughput studies, and therefore requires careful selection of lncRNA candidates. Genome-scale strategies for lncRNA CRISPR/Cas9 deletion are being developed [63], and variations in the ever-expanding CRISPR/Cas9 toolkit could help identify functional lncRNAs. Cas9 fused to repressors [64] or activators [65] now allows for the manipulation of expression levels at the loci themselves (figure 1). Using the former approach, researchers have identified human lncRNAs essential for cell growth in a diverse set of cell lines [64]. This technology is scalable and could help identify lncRNAs required in a variety of contexts by performing loss-of-function studies. The main limitation is that altering the chromatin state of the lncRNA promoter could directly affect nearby genes, complicating the interpretation of the phenotype.

### 3.2. Which long non-coding RNAs to study

The primary approach will depend on the biological question being asked. Some studies directly focus on a particular lncRNA of interest, while others aim at the unbiased identification of lncRNAs important for a process. Different strategies have been employed to select subsets of lncRNAs for study based on their expression level, dynamic regulation, tissue expression and even conservation.

Historically, highly abundant lncRNAs were chosen as representatives of this RNA class, with the hope of identifying possible mechanisms by which lncRNAs might act generally. This was, in part, due to experimental convenience and technical limitations, and this class includes the best characterized lncRNA to date, *Xist*, as well as *Malat1*, and *Neat1*. *Xist* orchestrates X chromosome inactivation. Expressed from the silenced allele, this lncRNA acts in *cis* to inactivate the expression of that X chromosome copy [66]. Focusing on a particular lncRNA has allowed researchers to channel all their efforts towards a mechanistic understanding of its mode of action, while also developing *in vivo* tools for its study. While *in vivo* functional validation is everyone's dream, placing all eggs in one basket is always risky and can lead to disappointing outcomes [20,52,53].

The opposite approach to studying a single lncRNA is genome-wide unbiased screening of lncRNAs. This can be an excellent filter to identify potential functional lncRNAs, building a resource for further mechanistic studies, although this approach is best suited to easily measured phenotypes, such as proliferation. Genome-wide screens have been used to identify lncRNAs essential for human cancer cell growth or survival [64], or those required in mouse ES cell self-renewal [47]. In ES cells, this approach identified *TUNA*, a lncRNA required for neural specification from ES cells that had already been described to have neural phenotype in zebrafish (named *Megamind*) [16], validating this strategy.

LncRNA annotation can be intersected with expression levels if one wishes to reduce the number of targeted lncRNAs. This can be necessary for more elaborate phenotypic assays that are not as scalable. A straightforward approach is to assess only the lncRNAs expressed in the cell type or tissue by setting some minimal expression cut-off [46]. However, for some strategies further reduction of lncRNA candidates is necessary. By analysing expression in different related tissues, especially in developmental systems, several groups have focused only on differentially regulated lncRNAs. The rationale behind this is that a transcript dynamically induced or silenced during a cell fate transition, for example, is more likely to be important for that process. Differential expression helped in the selection of candidates in epidermal differentiation [67,68], cardiac differentiation [69] and haematopoiesis [32].

Another extremely useful layer of filtering is evolutionary conservation. Although their sequence is not broadly conserved, lncRNAs can often be found in syntenic positions in different species. These ‘syntelogs’ can even share some small conserved domains [70]. This approach has been used to identify several conserved lncRNAs that act during zebrafish development [16] and drew researchers’ attention to *NORAD*, a conserved lncRNA in mammals that modulates Pumilio proteins [71,72]. Combined with differential expression, conservation can help focus the candidate list on lncRNAs with key functions in developmental or disease processes. As ‘syntenic conservation’ is a rather loose criterion and the presence of a lncRNA does not necessarily indicate that it will have the same function in a different organism, complementary strategies will be very helpful in identifying orthologous lncRNAs. If they are functionally conserved, lncRNA ‘syntelogs’ might share structure similarities, even if they do not share much sequence identity. Some studies have approached this by looking at predicted secondary RNA structure [73]. Although RNA structure predictions for long RNAs might not be particularly useful, new experimental approaches to globally identify structure features in lncRNAs could aid in this task [74].

When dealing with lncRNA annotation, it is important to be aware of the limitations. Although most assemblies set up stringent coding potential cut-offs, lncRNAs often contain very short open reading frames (ORFs). The functionality of these micropeptides is hard to assess unless one addresses it experimentally. Three different short proteins have been found to play a role in muscle function or regeneration [75–77], which emphasizes the importance of testing for RNA-mediated rather than protein-mediated effects.

Overall, the ability to modulate the expression of lncRNAs or disrupt it altogether now allows an assessment of lncRNA requirements in many developmental and cancer contexts. This, combined with some clever candidate selection strategies, has identified a number of lncRNAs important in these processes. The level of current mechanistic understanding for each of these lncRNAs is variable, yet the techniques available and being developed hint at a promising future.

## 4. Long non-coding RNAs shape development and cancer

Even for well-studied lncRNAs, our mechanistic understanding has deepened only in the last few years. The poster child for lncRNA researchers, *Xist*, orchestrates X chromosome inactivation. The functional properties of *Xist* and the order of events it directs have been known for decades (reviewed in [66]). However, it has taken until very recently to better understand the X inactivation at the molecular level. We now know the protein partners *Xist* requires for X chromosome silencing [78,79], how *Xist* spreads [80] and takes advantage of the chromosome's three-dimensional structure to initiate silencing [81], and how that chromosomal conformation changes during transcriptional silencing [82,83]. Additionally, this RNA is modified with N6-methyladenosines, which contribute to its transcriptional repressive activity [84]. *Xist* illustrates not only the detailed mechanistic understanding to which we can aspire for other lncRNAs of interest but also the tremendous amount of effort required to understand even a single lncRNA. Of note, *Xist* is also highly abundant when it is expressed, and that induction takes place in a cell type we can culture in large amounts (ES cells). Greater challenges can be expected with lncRNAs expressed to lower levels in very specific cell types.

**Figure 2.**
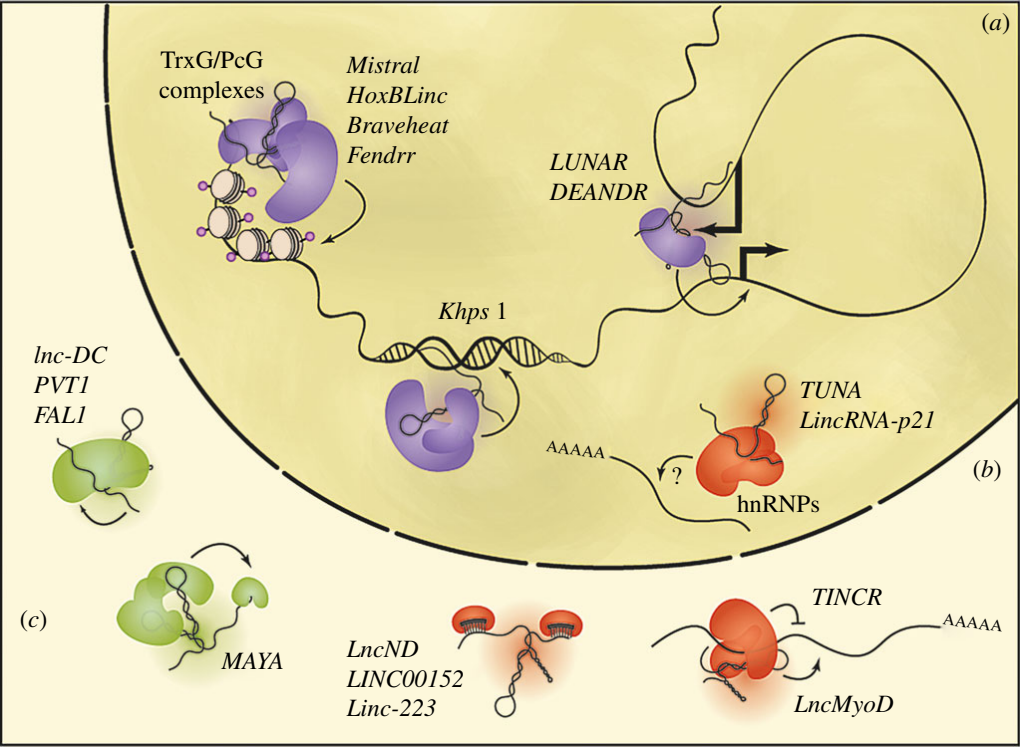
Themes in lncRNA functions. LncRNAs have been described to play multiple roles affecting gene expression at the transcriptional level via interactions with chromatin remodelling complexes, direct binding to the DNA as an RNA–DNA triplex, or facilitating chromatin looping (*a*), interacting with RNA processing machinery or affecting mRNA stability (*b*) or directly regulating protein function (*c*).

Some hints at how other lncRNAs exert their functions in development and cancer have been reported. Although the field is still maturing, lncRNAs have been described to play a myriad of roles, from regulating gene expression to regulating mRNA processing or affecting protein stability (figure 2). There are also several examples of lncRNA loci where the act of transcription but not the RNA itself seems to be of functional relevance [59,85] or where transcription is even dispensable [59]. Here, we focus on the RNAs themselves as the functional units.

### 4.1. Effects on chromatin and DNA interactions

Following *Xist*'s example, many researchers have focused on potential chromatin regulatory roles of lncRNAs. *Mistral* (*Mira*) is a lncRNA expressed in ES cells that is reported to interact with MLL1 to recruit this protein to *Hoxa6* and *Hoxa7*, leading to their activation. Consequently, siRNA-mediated knockdown of *Mistral* leads to reduced transcription of these genes and negation of the overall germ-cell specification programme [86]. Also expressed in ES cells, *HoxBlinc* binds to the same complex to promote *HoxB* transcription and mesoderm specification [87].

Two lncRNAs have been shown to be required for heart development. *Braveheart*, required for the production of contracting embryoid bodies from ES cells, interacts with the Polycomb factor SUZ12 [69], while *Fendrr*, a lncRNA essential for mouse development [17,18], binds to SUZ12 as well as EZH2 and WDR5 [17]. These interactions with members of the TrxG/MLL and Polycomb complexes place these lncRNAs in a position to direct chromatin modifications to particular DNA loci in a sequence-specific manner (figure 2*a*).

Following this hypothesized mechanism, it was shown that *Fendrr* interacts, at least *in vitro*, with the promoter regions of *Pitx2* and *Foxf1*, both of which are expressed during heart development [17]. In HeLa cells, *Khps1* regulates the promoter of its antisense gene, the proto-oncogene *SPHK1*, by forming a DNA–RNA triplex at its promoter while recruiting p300/CBP [88] (figure 2*a*). *TARID* also regulates its antisense gene, *TFC21*, by guiding GADD45A to the locus and promoting demethylation, which leads to gene activation. *TARID* and *TCF21* are silent and heavily covered by DNA methylation in non-small cell lung cancer (NSCLC), head and neck squamous cell carcinomas (HNSCC) and ovarian cancers (OVC) [49]. And although direct RNA–DNA binding has not been shown, lncRNA *SLNCR1* is required for bringing androgen receptor to the *MMP9* promoter, which increases *MMP9* expression and leads to melanoma invasion [89].

The ultraconserved lncRNA *Megamind*/*TUNA* showed brain development phenotypes upon knockdown in zebrafish [16] and is required for ES cell pluripotency and neuronal differentiation from mouse ES cells [47]. In the mouse, this lncRNA binds three RNA-binding proteins and interacts with the *Sox2* promoter [47]. Sox2 is a key transcription factor in neuronal differentiation, so its regulation could explain the resulting phenotype. And while the interaction seems to be indirect, *Dali* has been shown to localize globally to active promoters in the N2A neuronal differentiation model [90], as shown by CHART-seq [91].

Interaction with transcription factors themselves is another plausible mechanism to promote expression of specific gene programmes. *RMST*, for example, is a lncRNA up-regulated during neurogenesis that interacts with Sox2. Knockdown of *RMST* leads to a reduction in Sox2 ChIP-seq peaks in this model, which suggests that this lncRNA is somehow facilitating the binding of this transcription factor [92].

Additionally, *LUNAR* and *DEANR1* both seem to function by facilitating DNA looping between the lncRNA locus and their target gene to promote activation. *LUNAR* is a Notch-regulated lncRNA that activates the *IGFR1* gene in T-cell acute lymphoblastic leukaemia [51], while *DEANR1* functions via a similar mechanism in endoderm development, activating *FOXA2* expression through the recruitment of SMAD2/3 [93] (figure 2*a*).

### 4.2. Effects on mRNA stability and processing

Conceptually, the next level of regulation would be for lncRNAs to negatively or positively affect the stability or processing of coding mRNAs. By having cell-type or tumour-specific expression, lncRNAs would effectively control the output levels for these genes. In accord with this model, a few exemplar lncRNAs have been shown to interact with heterogeneous nuclear ribonucleoproteins (hnRNPs) (figure 2*b*). *LincRNA-p21*, induced in response to DNA damage, interacts with hnRNP-K, and it is regulated by p53. Knockdown of this lncRNA leads to up-regulation of genes normally repressed by p53 and also reduces apoptosis similarly to p53 knockdown [94]. This suggests a model whereby this lncRNA acts as a repressor of p53-dependent genes.

In neural differentiation, *Pnky* knockdown leads to progenitor expansion, and mass spectroscopy of lncRNA-interacting proteins followed by immunoblotting validation revealed PTBP1 as one of its interaction partners. Knockdown of this lncRNA leads to misexpression and altered splicing of many key genes [95]. Both PTPB1 and hnRNP-K also bind *TUNA* during *in vitro* neuronal differentiation [47]. Being highly expressed and broadly acting proteins, it is only reasonable to wonder whether these functions are truly specific. Only more detailed biochemical studies will be able to clarify this.

In a complementary approach to mass spectroscopy, protein microarrays identified STAU1 as the interacting partner of lncRNA *TINCR* [68]. Combined knockdown of *TINCR* and *STAU1* seems to affect the stability of important epidermal differentiation genes such as *Krt80* [68], which would explain its essential role in skin differentiation. During muscle differentiation, *LncMyoD* binds IGF2 mRNA-binding protein 2 (IMP2), which leads to enhanced translation of mRNAs involved in proliferation. Interestingly, this is a conserved lncRNA and the mouse and human sequence can rescue each other's knockdown [96] (figure 2*b*).

Rather than binding elements of the RNA processing machinery as a way to regulate the fate of coding mRNAs, other lncRNAs have been shown to act as endogenous competitors for microRNAs, thus dampening the silencing of microRNA targets. *LncND*, for example, is a primate-conserved lncRNA expressed in neural progenitors and down-regulated in neurons. This lncRNA competes for miR-143-3p, which would normally target Notch. Relieving Notch silencing promotes neuronal differentiation [97].

In the cancer context, *LINC00152* acts as an oncogenic lncRNA, competing with *HIF1-α* for miR-138. Expression of this lncRNA promotes invasion in gall bladder cancer [98]. As an additional example, *linc-223* would usually bind to miR-125-5p but it is down-regulated in acute myeloid leukaemia, leading to increased repression of *IRF4*, a target of miR-125-5p [99] (figure 2*b*).

The range of action of *lncARSR* extends even further because, apart from competing with mir-34 and miR-449 thus promoting stability of AXL and c-MET, this lncRNA can be packaged into exosomes to be secreted. Down-regulation of microRNA target genes renders renal cancer cells resistant to sunitinib, and secretion of the lncRNA can disseminate this property to neighbouring cells [50].

### 4.3. Effects on protein stability and function

Rather than affecting the mRNAs of genes important for differentiation or malignant proliferation, lncRNAs can also directly bind proteins essential for a signalling pathway and modulate their function. *Lnc-DC*, for example, is induced during dendritic differentiation from human monocytes. This lncRNA interacts with STAT3 and, when knocked down, leads to a reduction in Y705 phosphorylation of STAT3, decreasing its nuclear translocation. For that reason, expression of *lnc-DC* is required for differentiation of dendritic cells [100] (figure 2*c*).

Similar protein–lncRNA relationships have been observed in different cancer models. *FAL1* and *PVT1* are amplified in ovarian and breast cancer, respectively. *FAL1* associates with Bmi1, and *FAL1* knockdown leads to a reduction in Bmi1 levels and misregulation of large numbers of genes involved in cell cycle progression [101]. *PVT1* has a similar relationship with the oncogene C-MYC, promoting the stability of this protein [44] (figure 2*c*).

*lncRNA-LET* and *LINK-A* have opposing effects on *HIF1-α* in hepatocellular carcinoma. Enforced expression of *lncRNA-LET* leads to reduced *HIF1-α* and results in lower metastatic potential [102], while *LINK-A* interacts with tyrosine protein kinase 6 to promote stabilization of *HIF1-α* in triple negative breast cancer [103].

Other lncRNAs have specific roles in particular pathways, such as *MAYA*, a lncRNA recruited by HER3-ROR1 that then binds directly to NSUN6, preventing methylation of MST1. In breast cancer, MST1 is inactivated by this methylation resulting in YAP signalling target activation and increased bone metastasis [104] (figure 2*c*). *SAMMSON* is involved in regulating mitochondrial integrity by associating with p32. Knockdown of this lncRNA results in aberrant mitochondrial structures in melanoma, a cancer where *SAMMSON* is amplified [105].

By playing roles in essential cell functions, other lncRNAs also affect cancer progression. *NORAD* is a conserved lncRNA with repetitive regions that binds to PUMILIO proteins [71]. When this lncRNA is absent, PUMILIO proteins carry out their roles as negative regulators of mRNA stability and translation, and this results in aneuploidy [72]. *LINP1* interacts with Ku80 and DNA-PKcs, coordinating the non-homologous end-joining (NHEJ) pathway. Apart from providing essential functions for any cell, this pathway is particularly required in triple negative breast cancer [106].

## 5. Concluding remarks

lncRNAs are being heavily studied in the context of development and cancer, as their unique properties could allow them to interact with multiple proteins via three-dimensional structures and also recognize other nucleic acids by base pairing. Their specific expression during differentiation and disease places them in an ideal position to play key regulatory roles.

Because of the added complexity in studying lncRNA loci, a combination of genetic approaches is often required to distinguish between the function of the RNA molecule and the regulatory activity from a DNA element in that locus. Many examples have been described for lncRNAs interacting with chromatin, regulating genes at the RNA or protein level, or interfering globally with splicing. These functions are diverse and expand the original hypothesis of a nuclear-specific function for most lncRNAs. It was also proposed that lncRNAs would mostly act in *cis*, as their expression mirrored that of their neighbour genes [36,37,107,108]. Although that is the case for some examples, it does not seem to be a general rule [59].

LncRNAs are diverse molecules that are not likely to fit in one functional class. Consequently, we should start thinking of them more like proteins, some functioning in the nucleus [109], others acting in the cytoplasm and others supporting the structure of cells [110,111]. What is becoming clearer with the development of *in vivo* models and our expanding mechanistic understanding is that there are lncRNAs with essential functions in development and others required for cancer progression, taking this class of RNAs out of the ‘junk DNA’ category once and for all. Not every annotated lncRNA will have an RNA-mediated function—or a function at all—but identifying the biologically relevant ones and understanding their mechanisms will certainly be a hotbed for future study.

## Data accessibility

This article has no additional data.
